# Geriatrics in medical students’ curricula: questionnaire-based analysis

**DOI:** 10.1186/1756-0500-7-472

**Published:** 2014-07-25

**Authors:** Christoph HR Wiese, Kirstin Fragemann, Peter C Keil, Anika C Bundscherer, Nicole Lindenberg, Christoph L Lassen, Klara Markowski, Bernhard M Graf, Benedikt Trabold

**Affiliations:** 1 Department of Anesthesiology, University Medical Centre Regensburg, Franz-Josef-Strauß-Allee 11, Regensburg D-93053, Germany; 2 Centre for Education, University Medical Centre Regensburg, Franz-Josef-Strauß-Allee 11, Regensburg D-93053, Germany

## Abstract

**Background:**

Demographic development is accompanied by an increasingly aging society. Concerning medical education, the treatment of older people as well as the scientific research and exploration of ageing aspects in the coming years need to be considered. Aim of the study was to ascertain medical students’ knowledge, interest, and attitudes regarding older patients and geriatric medicine.

**Methods:**

Each participant completed a self-designed questionnaire. This questionnaire was based on three validated internationally recognised questionnaires (“Facts on Aging Quiz – FAQ”, “Expectations Regarding Aging – ERA” and the “Aging Semantic Differential – ASD”). The inquiry and survey were performed at the beginning of the summer term in 2012 at the University of Regensburg Medical School.

**Results:**

A total of n = 184/253 (72.7%) students participated in this survey. The results of the FAQ 25+ showed that respondents were able to answer an average of M = 20.4 of 36 questions (56.7%) correctly (Median, Md = 21; SD ±6.1). The personal attitudes and expectations of ageing averaged M = 41.2 points on the Likert-scale that ranged from 0 to 100 (Md = 40.4; SD ±13.7). Respondents’ attitudes towards the elderly (ASD 24) averaged M = 3.5 points on the Likert-scale (range 1–7, Md 3.6, SD ±0.8).

**Conclusions:**

In our investigation, medical students’ knowledge of ageing was comparable to previous surveys. Attitudes and expectations of ageing were more positive compared to previous studies. Overall, medical students expect markedly high cognitive capacities towards older people that can actively prevent cognitive impairment. However, medical students’ personal interest in medicine of ageing and older people seems to be rather slight.

## Background

Demographic development is accompanied by an increasing number of older people (adults aged 65 years and older) in society; decreasing birth rates, increasing life expectancy and a total “Change in the age structure towards older age groups” [1,2] are contributors to this fact. Thus, an ageing society is not a phenomenon that will be an issue in the distant future but already exists in the economy and medicine [1,3,4]. Due to increased life expectancy, hospital care will most likely increase over the next 20 years, especially in an elderly population [5].

Both, the treatment of older people and the scientific research and exploration of the aging process need to be considered in the coming years [6]. However, the recognition of the complexity of medical treatment in older patients appears to have only just begun. Since 2003, German medical universities have included geriatric education as a part of their curriculum (known as Q7) [7,8]. The education curriculum “Q7” is intended to prepare medical students for the treatment of older people in generally (for example pharmacological therapy in older people, physiology in older people, pathophysiology in older people, non-pharmacological therapies in older people) [8]. Overall, compared to the average population, medical students display an increased negative attitude towards older people [9]. Such attitudes, as well as a lack of interest in older people, have been described in several international and national investigations [9-13]. For this reason, it is of particular importance that some necessary features are taught during medical education related to the diagnosis and treatment of older people [14]. International studies were able to demonstrate a correlation between medical students’ knowledge, attitudes, experience and interest regarding ageing and older people [14-18].

Actually, there is no uniform policy for the implementation for Q7 in German medical universities [8]. A similar situation, with respect to curriculum content, in geriatric medicine was described for the US in 1983/2005 and for Singapore in 2009 [15,18,19].

### Aim of the study

The aim of the study was to ascertain medical students’ (7th, 8th, and 10th study semester) attitudes toward older people specifically, with differences in these attitudes explored on the basis of gender, semester, spirituality, intended medical specialty and prior medical education in geriatric medicine.

## Methods

### Study design and study instruments

The present study is comprised of an empirical analysis of a cross-sectional study on the attitude of medical students towards geriatrics, based on Q7. A self-developed national questionnaire based on validated international instruments (“Facts in Aging Quiz – FAQ”, “Expectations Regarding Aging – ERA”, “Aging Semantic Differential – ASD”) and our prior experience was answered by medical students [20-23]. The present study examined three factors in accordance with Tews (1991) that influence the development and severity of age images (knowledge and understanding of ageing and one’s own expectations with respect to age and aging) and correlated these factors with the independent variables [24]. The sample group chosen in our investigation is comparable to those found at other medical universities in Germany. Therefore, the conclusions drawn may be representative of medical students in Germany.

The meaning of “attitude” in the present investigation was to get to know medical students predispositions and tendencies towards older people. In our context, attitudes may be influenced by emotions or feelings, cognitive beliefs, religion, and/or experiences concerning older people (therapeutically or personal).

The survey was carried out using a standardised, self-report questionnaire. When designing the questionnaire, the following aspects were considered: (1) the phrasing of questions and answers, (2) sample analysis, (3) the volume of the control sample, (4) analysis and results, and (5) the interpretation of data. When phrasing the questions and answers, the focus was on a simple and commonly understandable formulation. Participants were informed that the term “older person” refers to people 65-years-old and above, according to the WHO definition. However, this term may also refer to people within the nationally common retirement age (currently 65 years in Germany) [5]. The questionnaire consisted of questions from the following categories: free answers, Likert scale (0–100 or 0–7, respectively) and multiple choice questions (right/wrong, predetermined answers and free text responses) and contained a total of 94 items. The contents of the questionnaire were the following:

1. Demographic data (e.g., age, gender, study year, living conditions, religiousness) – free text responses, Multiple Choice, Likert Scale 0-100

2. Content cross-sectional area 7 (Q7) – free text responses, Multiple Choice

3. Needs of students in the teaching of cross-sectional area 7 (e.g., scope, content, examples) – Likert Scale 0-100

4. Substantive meaning and the evaluation of different topics in cross-sectional area 7 (e.g., basic knowledge, clinical issues, health economics, medical specialty) – Likert Scale 0-100

5. Modification of the German translation of the “Facts on Aging Quiz – FAQ 25” (Questions out of FAQ 25) – true/false answers

6. Modification of the German translation of “Expectations Regarding Aging –ERA12/ERA 35” (questions out of ERA 12) – Likert Scale 0-100

7. Modification of the German translation “Aging Semantic Differential – ASD 24” (questions out of ASD 24) Likert Scale 1-7

In the following part, we show some examples of questions which were used in the questionnaire [22,25,26]:

1. Please define your interest concerning Q7 (Rating scale 0-100).

2. Do you think that the medical study of ageing is necessary (Rating scale 0-100)?

3. Please rate the following statements (e.g. findings to your own settings to ageing, knowledge of the fundamentals of ageing, knowledge of clinical issues and treatment options as well as prevention and ethical/legal principles in ageing) concerning a possible curriculum "medicine of ageing and older people” (Rating scale 0-100).

4. Questions of the German translation of the “Facts on Aging Quiz – FAQ 25” – (true/false, e.g. “the majority of old people are senile”, “physical strength tends to decline”, “about 80% of the aged are healthy enough to carry out their normal activities”)

5. Questions of the German translation of “Expectations Regarding Aging –ERA12/ERA 35” (Likert Scale 0-100, e.g. “older people have to reduce their claims”)

6. Questions of the German translation “Aging Semantic Differential – ASD 24” (Likert Scale 1-7, e.g. “older people are wise → foolish, kind → unkind, friendly → unfriendly, optimistic → pessimistic”)

### Investigation implementation

Questionnaires were distributed at the beginning of each lecture. Participation was voluntary, and the fill out of the questionnaire was considered as agreement to participate. There was no subsequent publication of the used questionnaires which were used in the present investigation.

### Interest in geriatric medicine

A total of 15 items on the questionnaire were content-related questions referring to “Medicine in ageing and older people”. Furthermore, the participants’ interest in geriatric medicine (Likert scale 0 = not important – 100 very important), rating of a sample course-model (Likert scale 0 = not important – 100 very important) and possible desires for Q7 specialisation (Likert scale 0 = not important – 100 very important) were evaluated.

The **Expectations Regarding Aging 12 (ERA 12)** measures the respondent’s attitude towards age via 12 statements. Originally, the ERA measured 38 items with a four-level Likert scale [20,22]. The ERA 12 is considered to be a global test with a consistent score over all 12 items. However, it is divided into three sub-scales (physical, mental and cognitive). Lower values in each of the global and sub-scores show expectations that physical and mental health as well as cognitive capabilities will reduce with age. Higher values suggest an active and positive attitude toward ageing [20].

The **Facts in Aging Quiz (FAQ)** measures common and specific knowledge on ageing and older people using up to 50 items (right/wrong). A correctly answered statement is rated with one point. In total, a calculation of cumulative values is performed [25]. For the present study, the questionnaire was adapted to national particularities.

### Aging semantic differential 24 (ASD24)

The **ASD 24** measures the respondent’s general attitude towards older people [23]. Twenty-four seven-stepped pairs with a Likert scale (1–7) are presented. The number four represents the mid-point to provide the possibility of a neutral rating to the participant.

### Time of study, participants and variables

The inquiry and survey were performed at the beginning of the summer term of 2012 (May 2012) at the University of Regensburg Medical School. All students within the 7th, 8th and 10th semester were included. The 9th semester was excluded because the cross-sectional Q7 “Medicine of ageing and older people” is integrated into this semester. This coursework may have influenced the students’ attitude, and this confounding factor was avoided for the survey. The fact that students within the 10th semester already completed the Q7 must be mentioned because there may be some implications for interpretation of our results.

For the survey, the following independent variables were defined:

(1) Age

(2) Gender

(3) Living situation

(4) Spirituality

(5) Training request

The following dependent variables were defined:

(1) Interest in medicine of ageing and older patients

(2) Evaluation of Q7 “Medicine of ageing and the older people” in reference to meaningfulness, content and structure

(3) Questionnaire “FAQ 25+”

(4) Questionnaire “ERA 12”

(5) Questionnaire “ASD 24”

A positive selection bias could be avoided by including all students attending the lecture at the time of the survey. The students had no prior knowledge of the survey; therefore, an absence because of the study could be eliminated.

Questions of the present investigation

• What predictors are available to evaluate students’ attitudes to “medicine of older people” during university studies?

• What knowledge on ageing and older people do students have?

• What attitudes do students of human medicine have towards ageing?

• What attitudes do students of human medicine have towards older people?

### Additional data

In addition to the above data, socio-demographic variables (semester of study, gender, age, living-situation, career, aspiration, faith and religion) as well as variables relating to Q7 “Medicine of ageing and older people”, assessment of a paradigm regarding organisation (in the style of the medical faculty of the Köln University Medical School’s organisation) and previous curriculum content related to medicine of older people during the course of studies were evaluated [27]. Calculations of point-grade-assignments were performed according to the Chair in Business Informatics of the University of Regensburg’s recommendations [28].

### Data collection and statistical analysis

All questionnaires returned were included for evaluation. Data protection regulations in accordance with the Declaration of Helsinki were adhered to by the pseudonymisation and encryption of the data. The data collection was acquired primarily via the written questionnaires and secondarily via the generation and transfer of a spreadsheet to Microsoft Excel (Vs. 2010, Microsoft. Inc. USA). A single person performed the data transfer using defined coding parameters. The spreadsheet was generated in MS Excel was then converted to the statistics software SPSS Statistics for Windows (Vs. 19.0, SPSS Inc. Chicago, Illinois USA). Descriptive data were presented as absolute values, percentages and partly as confidence intervals, means and/or medians.

A comparable statistic in reference to the questions of the survey was performed via *t*-test for independent samples, the Kruskal-Wallis Test for inter-group comparison and the post-hoc Bonferroni test for intra-group evaluation and multiple-testing correction.

The correction of p-values was performed with the Bonferroni-Holmes test for multiple comparisons when necessary. The Pearson chi-squared test and Bonferroni test for multiple comparisons were amongst other tests used for statistical analysis of possible significance between the three study semesters and the differences relating to the variable “gender”. Differences within the groups and the mentioned variables furthermore were calculated using one-way ANOVA (“ANalysis Of VAriance”). Differences within the groups compared with the other groups were calculated using two-way ANOVA.

*T*-test for independent samples, significant differences, and possible significance of dependent variables regarding the independent variables between the groups was used for calculation. P-values <0.05 were defined as being statistically significant in all statistical calculations. Correlations were statistically evaluated for the relationship between two variables using the rank-correlation coefficient “Spearman’s Rho” (r).

The University of Regensburg’s ethics committee was informed of the study. Due to the study design, the University of Regensburg’s ethics committee approved the investigation (No. 12-160-0177, Ethics Committee, University of Regensburg, Germany).

## Results

### Demographics

A total of n = 184/253 (total/returned: 72.7%) students of Human medicine at the University of Regensburg Medical School participated in this survey. Those participants are distributed as follows:

(1) 7th semester of study: 63/93 students (participation rate of this semester: 67.7%)

(2) 8th semester of study: 72/82 students (participation rate of this semester: 87.8%) and

(3) 10th semester of study: 49/78 students (participation rate of this semester: 62.8%).

All questionnaires returned (100% of students attendant during survey) were integrated into the study (see Table 1). There was no statistically significant difference (p > 0.05) of the independent variables “number of students per semester” and “age” and no significant differences of the independent variables “gender”, “living-situation”, “career aspiration”, “religion” and “faith”. Therefore, all participants were comparable with regards to the demographic data in correlation with the dependent variables.

**Table 1:** Demographic data of the participants (data in absolute values, percentage/%, median score (Md) and/or standard deviation/SD); “*”p < 0.05 (statistical significance in the intra-group evaluation); “**”p < 0.05% **(statistical significance in relation to all respondents); “°°”p < 0.05**% **(statistical significance in relation to the intra-group-evaluation and all respondents)**

**Item**	**Total**	**7th semester**	**8th semester**	**10th semester**
	**n (%)**	**n (%)**	**n (%)**	**n (%)**
**Study year**	184 (100)	63 (34.2)	72 (39.1)	49 (26.6)
**Gender**				
male	61 (33.2)	16 (25.4)*	29 (40.3)*	16 (32.7)
female	123 (66.8)	47 (74.6)*	43 (59.7)	33 (67.3)
**Age (y)**				
Md	27.5	27	26	28
SD	±3.26	±3.13	±3.27	±3.24
min./max.	21/46	22/46	21/30	23/31
**Living situation (n = 184)**				
alone	72 (39.1)	31 (49.2)*	24 (33.3)	17 (34.7)
with partner	29 (15.8)	7 (11.1)	7 (9.7)	15 (30.6)*
Flat share	74 (40.2)	22 (34.9)	35 (48.6)	17 (34.7)
with parents	6 (3.3)	2 (3.2)	4 (5.6)	0
Other	3 (1.6)	1 (1.6)	2 (2.8)	0
**Training request (n = 184)**				
General practitioner/Internal Medicine	33 (17.9)	9 (14.3)	19 (26.4)*	6 (12.2)°°
Anaesthesiology				
Surgery	12 (6.5)	4 (6.3)	4 (5.6)	4 (8.2)
Paediatrics	19 (10.3)	3 (4.8)°°	9 (12.5)	7 (14.3)
Other	11 (6.1)	4 (6.3)	4 (5.6)	3 (6.1)
Unknown	39 (21.2)	14 (22.2)	12 (16.7)	12 (24.5)
	70 (38)	29 (46)*	24 (33.3)	17 (34.7)
**Religion (n = 184)**				
Roman Catholic	112 (60.9)	28 (44.4)°°	51 (70.8)°°	33 (67.3)*
Protestant/Lutheran	48 (26.1)	20 (31.7)*	16 (22.2)*	12 (24.5)
Muslim	3 (1.6)	2 (3.2)	1 (1.4)	0
Other	6 (3.3)	4 (6.3)	2 (2.8)	0
None	15 (8.2)	9 (14.3)*	2 (2.8)°°	4 (8.2)
**Belief (n = 184)**				
Md	51.7	48.2	53.8	51.5
SD	±30.5	±29.3	±31.5	±30.8
Likert Scale min./max.	0/100	0/100	0/100	0/100

The median score in relation to the personal interest in “Medicine of ageing and older people” was 51.9 (SD ± 29.9. min./max. 0/100). The rating of Q7 “Medicine of ageing and older people” as being reasonable was mean = 53.6 (Md 50. SD ± 23.3. min./max. 0–100). The mean score for the presented course-model was rated 58.2 (Likert-scale “not reasonable” 0 to “very reasonable” 100; Md = 60, SD ± 26.2; range 0–100).

We found a statistically significant desire of respondents for a better/extended amount of structure and organisation and an extended amount of practical training (p < 0.05). Further content-related desires regarding the optimisation of Q7 are shown in Table 2 (Likert-scale “not important = 0” to “very important = 100”).

**Table 2:** Rating of desires regarding the optimisation of Q7 by respondents (all data are presented as mean score (M), median score (Md) and standard deviation (SD) and min./max.; “*”significance p < 0.05)

	**Awareness of personal attitude**	**Knowledge of the principles of ageing**	**Knowledge of clinical questions**	**Health economics**	**Practical skills**
M	48.8	68.2*	68.1*	51.6	65.1*
Md	50	70*	70*	50	70*
SD (±)	28.6	22.5	20.9	25.3	22.5
min./max.	0/100	0/100	5/100	0/100	0/100

### Analysis of (1) “semester of studies”, (2) “age, gender, living situation, and faith” and (3) career aspiration and religion vs. attitude toward medicine of older people

(1) We found statistically significant differences (multi-factor-analysis) when comparing the year of study (7th vs. 8th vs. 10th semester) with the respondents’ attitude towards (1) usefulness of age-medicine-related contents during education (*7th vs. 8th semester: p = 0.007; 7th vs. 10th semester: p = 0.022*). (2) structural/organisational improvements of Q7 (*7th vs. 10th semester: p < 0.0001; 8th vs. 10th semester: p < 0.0001*), (3) increased practical fraction (*7th vs. 10th semester: p < 0.0001; 8th vs. 10th semester p < 0.0001*) and (4) increased number of seminars during the course (*7th vs. 10th semester: p < 0.0001; 8th vs. 10th semester: p < 0.0001*).

The introduced course model consisting of theoretical, practical and eligible components to expand Q7 was significantly more often rated to be more feasible and meaningful by students from the 10th semester than by those of the other semesters integrated into the survey (p = 0.042).

(2) The entire data set was homogenous concerning the variable “age”. Therefore, the result is considered to be of limited value (p > 0.05). We observed comparable values regarding the independent variables “gender” and “living situation”; therefore, we found no statistically significant differences in the attitude towards medicine of older people during the course of study (p > 0.05). Furthermore, a statistically significant majority of respondents that characterised themselves as having “faith” showed an increased interest in “Medicine of ageing and older people” than those who characterised themselves as having less “faith” (p < 0.05).

(3) Definitive career aspiration plans were not specified by more than 50% of all respondents at the time of the survey (see Table 1). When sub-classifying respondents into the categories “existing career aspiration”, “not existing career aspiration” and “career aspiration unknown”, a statistically significant majority of respondents with an already existing career aspiration had a desire for an increased number of lectures and seminars on Q7 (p = 0.08 resp. p = 0.037). Religion did not appear to be a valuable parameter to generate respondent’s attitudes toward medicine of older people.

### Analysis of (1) “gender”, “living situation”, and “semester of studies” and (2) “personal interest in medicine of ageing and older people” vs. the results of ERA 12, FAQ 25+, and ASD 24

(1) The respondent’s gender and their living situation did not correlate with the student’s attitude toward the results of ERA 12, FAQ 25+, and ASD 24 in general (p > 0.05). However, we were only able to find statistically significant differences between the different semesters for the statements (1) “I’m expecting to spend less time with family and friends the older I get” (p = 0.032) and (2) “Depression is a completely normal component/process of ageing” (p = 0.049).

(2) Respondents (n = 81) rated their personal interest as being “low” or “very low”, while n = 44 (24%) rated it “high” or “very high”. No statistically significant differences between the groups were observed (p > 0.05).

### Knowledge about ageing and older people (FAQ 25+) and respondents’ attitudes and expectations of ageing (ERA 12)

The mean score for the results of the FAQ 25+ showed that respondents were able to answer 20.4 of 36 questions (56.7%) correctly (Median, Md = 21; SD ±6.1). According to the assessment criteria 75% of the respondents may have pass such a test. Further correlations can be found between the correct estimation that “old people certainly are capable of learning new facts” (92.9%) and the estimation that “it is very possible to counteract age-associated mental impairment” (r = 0.8).

The personal attitudes and expectations of ageing averaged 41.2 points on the Likert-scale ranging from 0 to 100 (Md = 40.4; SD ±13.7). Higher values of the three sub-scales (physical and mental health and cognitive function, see Table 3) on the Likert-scale imply a positive attitude towards one’s own ageing.

**Table 3:** ERA-12 scores of the study participants (mean and standard deviation)

**ERA-12 Score (mean score and standard deviation)**	
**Total ERA-12**	41.2 (±13.7)
**Physical Health**	43 (±18.3)
**Mental Health**	37 (±17.5)
**Cognitive Function**	46.5 (±17.7)

Most notably, the phrases “repairs will be necessary”, “being alone will increase”, “forgetfulness will increase” and “memory in general will diminish” with age were rated significantly higher by our respondents (p < 0.05). The statement that a “certain mental reduction cannot be avoided” was assessed very critical by our respondents. It is evident that on one hand, an increased mental reduction is expected with age, while on the other hand, the possibility of counteracting this process is very likely.

### Respondents’ attitudes towards older people (ASD 24) and correlation between knowledge (FAQ 25+) and respondents’ attitudes towards older people (ASD 24)

A statistical comparison (two-way multi-variable ANOVA) between the independent variables and the dependent variable “ASD 24” showed no statistically significant differences between the groups. Respondents’ attitudes towards older people averaged 3.5 points on the Likert-scale (range 1–7, Md = 3.6, SD ±0.8).

No statistically significant correlations between the entirety of factors could be observed (in each case p = 0.07 and r = 0.13, “respectively”, r < 0.3). It was demonstrated that a higher standard of knowledge tended to correlate with a positive image of older people (correlation between FAQ 25+ and ASD 24 in general). However, there were individual aspects which statistically significant showed that a higher level of knowledge correlates with more positive attitudes concerning older people (for example older people are trustfully, hopefully, selfless, happy, likely; p < 0.05). Moreover, high knowledge correlates with positive attitudes regarding the assessment of physically changes in older people (r = 0.8).

## Discussion

The general attitude of students towards older people, their own age and ageing is internationally described as being rather positive and as well as negative [9,15,16,21,29,30]. Overall, it has been shown that the majority of medical students cannot imagine working in geriatric medicine [9,14,17,31]. The present study was able to validate those findings of earlier investigations. The main aspect of this study, in contrast to multiple previous studies, was the focus on the assessment of independent variables (such as gender, career aspiration, religion, and faith) regarding the students’ attitudes towards geriatrics and older people as a field of education during their course of study. Therefore, the focus and results on general attitudes towards ageing and older people were not comparable with previous investigations in all aspects. The assessment of the respondents’ general attitude towards older people showed a rather positive image. Despite partly contradictory studies, these attitudes could be confirmed by several other studies and therefore correspond with the present study [9,31]. Overall, in terms of attitude, it remains to be determined whether the students are negative towards older people in general, personal ageing and/or towards the treatment of older people in general. There seem to be obvious differences in this context. Although we were unable to correlate “religion” with “attitudes to older people”, it is necessary to mention that the sample of the present study was mainly composed of participants who were catholic (as a phenomenon in Bavaria, Germany). Therefore, it may be that if more religions will be integrated to other investigations, religion may have a greater role to play. Concerning this fact the present study may not be representative for the situation in Germany in general.

Earlier studies had the conclusion that not only education and knowledge transfer but especially the personal attitude towards age, experiences with older people, knowledge of ageing and attitudes towards older people correlate positively with future behaviour and medical therapy towards older patients [9,14,15]. The present investigation was able to validate those results only partially. Some correlations could be shown not only to be statistically significant but also to be partially clinically relevant. These results contradict past studies [14,21].

Earlier investigations identified sociodemographic factors (e.g. gender and nationality) as predictors of students’ attitudes towards older people/patients. The present investigation could not detect such factors compared with the results of those studies [6,9,31]. The respondents’ personal career desires had a significant influence on their attitudes towards geriatric medicine, and an existing educational desire led to the desire for increased lectures and seminars regarding Q7. Participants who already had an existing educational desire or already started their specialist training demonstrated an increased desire for geriatric content during their course of study and had a rather positive image of older people [15,16,21,32].

At the surveyed medical school, the cross-sectional area 7 (Q7) is taught during block lectures and seminars within the 9th semester. In addition, courses on medical characteristics of older people are conducted during further block lectures, lectures and seminars (e.g., internal medicine, neurology, and surgery). Altogether, a practice-oriented concept has been rated significantly more feasible and meaningful by students of the 10th semester than by those from the other semesters (p < 0.042). This fact correlates with the desire for increased amounts of practical components within geriatrics education. Therefore, target-oriented workshops for future practitioners (students and residents) are provided at the annual conference of the German Association for Geriatric Medicine (DGG) to increase interest in the field by the practice-oriented transfer of knowledge. The fact that students within the 10th semester already completed the Q7 must be mentioned at this time again, because there may be some implications for interpretation of our results.

Concerning future work in geriatric medicine, none of our respondents stated a possibility of medical work as personally relevant. Approximately 40% of all respondents had no desire for future specialist training. These findings are contrary to previous data from Chua et al. in 2008 [32], where 35% of respondents (1st and 2nd semester) stated that they had a possible desire to work in geriatrics in the future. Voogt et al. (2008) also found that most students did not have future aspirations to work in geriatrics; therefore, those results are congruent with the present data [14]. Nevertheless, in this study, the proportion of students who could at least imagine future work in the field of geriatrics (15%) was relatively high compared with the present data [14]. In the literature, at least a few students plan to work in the field of geriatrics in the future [32]. In general, the results of this investigation were not able to detect a statistically significant correlation between personal attitude, knowledge and attitude towards older people on the basis of this variable.

Students who already had undergone geriatric education could have been expected to have superior knowledge in the field [33]. In general, a correlation between knowledge and attitude towards older people could also not be observed in this investigation. However, single aspects were able to show a correlation between higher knowledge and attitudes regarding older people (r = 0.9). Overall, education and knowledge may improve positive attitudes to older patients as earlier studies recommended as well [14,15,32]. Nevertheless, it is recommended in the literature that knowledge can be gained by enhancing education for all occupational groups through contact with older people, achieving a general attitude optimisation [33,34]. In Germany, the transfer of knowledge in geriatrics is not achieved as a specialty but rather in terms of a so-called cross-sectional area [34]. Renteln-Kruse et al. (2009) published the first national evaluation of geriatrics, which showed a wide acceptance in terms of content-based and formal design [35]. This acceptance will have to be critically questioned, and there will be limited objectivity regarding German universities. However, it shows the difference between the acceptance of courses and the cognition of the meaningfulness of geriatric content in the study of human medicine. Therefore, our respondents seem to have a relatively small interest in geriatrics; a teaching evaluation, however, could show more positive results. However, previous experiences show no increased knowledge based on a better teaching evaluation [29].

The pure transfer of knowledge did not correlate with students’ attitudes towards older people in previous surveys. The goal of medical education is based on the transfer or theoretical knowledge as well as practical skills to enable students to implement the theoretical knowledge [29]. An improved education using different didactic options generally leads to an improved competence and an enhanced attitude towards professional conduct, therapy and attitude towards patients [29]. The general image of old age was rather positive in the present survey as well. However, the personal interest in the medicine of ageing and older people was rated as being rather slight.

Concerning the ERA-12, the results correspond with previous data; although data from the present study tend to be higher than those from comparable studies [9,20]. Its relevance for daily practice cannot be judged at this point. No statistically relevant differences between the semesters could be observed, which corresponds very well with the findings of Klaghofer et al. (2009) and therefore implies an increased explicability [9]. On the contrary, it was not possible to affirm the fact that increased personal expectations of ageing correspond with a positive image of age [9]. Therefore, the model of the image of old age can be confirmed as a trend but not statistically significant [24]. Also, when comparing single variables, such as “gender”, “living situation”, “semester of studies”, “faith” and “religion” as well as personal interest in geriatrics and assessment of the meaningfulness of geriatrics during the course of studies, no statistically significant factors and therefore no factors that are able to detect the image of old age could be found. Overall, the ERA 12 represents a probate and reliable instrument to detect attitudes towards ageing. This was also confirmed by Joshi et al. (2010). However, the desired and intended influence on the image of old age could not be predicted by ERA 12 in this survey [20].

Overall, the respondents from the present study showed a rather positive image of older people. A positive image of older people could also be affirmed by previous surveys [9,14,31]. It was not possible to detect correlations between desires and interests regarding structural and content-based aspects of geriatrics during the course of study. We have no knowledge of previous comparable studies; therefore, further studies are necessary. Overall, no factor could be found that could eventually be used to predict the image of old age, which corresponds very well with previous surveys [9,31]. It can only be assumed that this result is due to the fact that most respondents were in their early twenties and that their grandparents can be assigned to a rather vital generation; therefore, the respondents have a rather positive image of old age. However, they did not get in touch with multi-morbid patients during their education and therefore have not observed the drawbacks of ageing with increased medical possibilities.

### Limitations of the study

This investigation has some limitations due to the study design and the materials used. A major limitation is the chosen study design. A questionnaire-based survey is limited by the questionnaire itself because many aspects cannot be evaluated in a single survey. Furthermore, by providing an excessive number of questions, the respondents’ patience can be tested, reducing the accuracy of the answers.

Second, a self-constructed questionnaire poses the danger of introducing problems and biases by the authors. Possible deficits are only theoretical and were not recorded in its practical expression, presentation and evaluation and discussion. Thus, a potential clinical consequence for the practical care and the practical handling of the patient as a whole remains unconsidered. In addition, the further development of the respondents remains to be observed in their further training and can be assessed in only a limited way at this point in terms of future jobs. Overall, the practical clinical relevance of a purely theoretical survey is not clearly assessed (including the data and knowledge survey). The survey as such can lead to possible consequences for the respondents, as content may be processed in the sequence due to dealing with the issue. Thus, a positive change in the clinical setting is possible. The knowledge gaps that are collected by questionnaires may also be assessed only as references but not as unique circumstances.

Another limitation was the fact that no practical options for action in specific exemplary scenarios were questioned in the investigation. A possible empowerment of participants could therefore be checked either theoretically or practically.

## Conclusion

The present study aimed to epidemiologically evaluate students’ attitudes towards geriatric content during the course of study, aspects of knowledge, attitudes towards personal ageing and attitudes towards older people or patients. Overall, it was not possible to determine predictors with which students’ attitudes towards the content and structure of geriatric training can be assessed. Guidelines for learning objectives and skills profiles as well as an increased practical proportion during Q7 tend to be desired and useful. Students’ knowledge on age and ageing were shown to be comparable with previous investigations.

Attitudes and expectations of ageing were more positive compared with previous studies. Above all, students expect markedly high cognitive capacities at an older age and believe that individuals can actively prevent cognitive impairment. In this respect, ageing is not considered to be a negative occurrence.

The image of old age was generally positive; therefore, there was an incongruity in the interest in geriatric medicine. The positive image of old age should be an occasion to improve the apparently unpopular subject of geriatrics by means of the cross-sectional area 7 and to reinforce this view by multiple contacts with patients and simulations. Yet, the difference between perfect teaching concepts and the obviously limited resource of the “teacher” must be kept in mind. Such an improvement definitely has to be aimed also toward the present results because the subject of geriatric medicine will personally concern each and every one of us in the future. The special importance of this cross-sectional medical education should be recognised in the present and in the future.

## Competing interests

The corresponding author and co-authors confirm that we have no connections to any of the companies whose products are mentioned in the article, or with any company that sells competing products. The manuscript contains parts of a master’s thesis (CHRW; Course of Study “Master of Health Business Administration”, University of Nürnberg, Germany).

## Authors’ contributions

CHRW, KF, PCK, AB and KM participated in designing the study. CHRW, NL and KM participated in collecting and entering the data. BMG, CLL and BT supported in editing the manuscript. PCK co-wrote the manuscript and added important comments to the paper. All authors read and approved the final manuscript.
